# circTNFRSF21, a newly identified circular RNA promotes endometrial carcinoma pathogenesis through regulating miR-1227-MAPK13/ATF2 axis

**DOI:** 10.18632/aging.103037

**Published:** 2020-04-16

**Authors:** Yun Liu, Yue Chang, Yixuan Cai

**Affiliations:** 1 Department of Obstetrics and Gynecology, Beijing Friendship Hospital Affiliated to Capital Medical University, Beijing, China

**Keywords:** circular RNA, endometrial carcinoma, miR-1227, MAPK13, ATF2

## Abstract

Background: Circular RNA is a type of non-coding RNA with great potential in regulating gene expression and associated with disease progression. However, the role of circular RNA in endometrial carcinoma (EC) remains largely unknown.

Results: In this study, we found that circTNFRSF21 was highly expressed in EC cells and tumor tissues. In vitro and in vivo results showed that circTNFRSF21 was linked to increased EC cell growth and EC xenografts formation in nude mice. Mechanically, we showed that circTNFRSF21 acts as a sponge of miR-1227 in EC cells to rescue MAPK13/ATF2 signaling pathway activity.

Conclusions: Our studies suggested that in the EC, circTNFRSF21 promotes EC formation through downregulating miR-1227 expression and activating MAPK13/ATF2 signaling pathway. These findings provide strong evidence that circTNFRSF21-miR-1227-MAPK13/ATF2 axis is a promising target for EC treatment.

Methods: qRT-PCR was used to detect circTNFRSF21expression in EC patients and EC cell lines. Cell growth, cell colony formation, cell apoptosis, cell cycle progression, and in vivo tumor formation assays were used to evaluate the roles of circTNFRSF21 in EC. Western blot, luciferase assay, RNA pull-down, siRNA knockdown, and CRISPR gene knock out assays were applied to study the mechanisms through which circTNFRSF21 regulates EC formation.

## INTRODUCTION

Endometrial carcinoma (EC) is the most common gynecologic malignancy of the female reproductive system [1]. According to clinical statistics, the newly diagnosed EC cases have risen from 60,050 to 61,880 with the mortality rate elevated from 10,470 to 12,160 between 2016 and 2019 in the United States [2] In general, EC can be divided into two different categories: estrogen-dependent type I and estrogen-independent type II [3–5]. Type I EC was characterized by estrogen mediated with high rates of PTEN and K-ras loss or mutation [6–9]. Most type I EC patients exhibit low grade adenocarcinomas with relatively good survival rates [10, 11]. In contrast, type 2 EC patients were mostly in the higher-grade adenocarcinomas when diagnosed. Type 2 EC may arise from atrophic endometrium and is usually associated with a lack of estrogen. Due to the highly aggressive cancerous tissue, the prognosis of type 2 is poor.

Non-coding RNAs are groups of RNA that do not encode any proteins. Many non-coding RNAs including long non-coding RNAs and microRNAs (miRNAs) have shown to play critical roles in biological processes including cellular differentiation, tissue homeostasis as well as in the development of diseases including cancers. Circular RNA (circRNA) is a group of newly discovered non-coding RNAs. In recent years, an explosive growth of publication has focused on characterizing circRNA, highlighting circRNA may play an important in cell biology. Different to other RNAs, circRNA consists of a continuous closed loop structure generated from its precursor mRNA through back splicing [12, 13]. Recently, emerging studies have shown that circRNA may function as a sponge of miRNA and affect miRNA targeted gene activity. For example, CDR1as was predicted to have 73 binding sites for miR-7 and one binding site for miR-671, knockdown of CDR1as significantly decreased miR-7 level and increased miR-671 expression [14]. Moreover, in the brains of CDR1as-knockout mice, several miR-7 targeted genes were also downregulated.

The advancement of next generation sequencing technology greatly facilitated the studies of circRNA in cancers [15, 16]. Through pair end sequencing, many differentially expressed circRNAs between cancers and normal tissues have been identified. Those circRNAs are potential biomarkers or molecular targets for cancer diagnosis and treatment.

Until now, the role of circRNA in EC remains largely unknown. Recent studies with genome-wide sequencing showed that hsa_circ_0001610 derived from TNFRSF21 was greatly upregulated in grade 3 EC than their adjacent non-cancerous endometrial tissue [17]. However, the biological property and significance of circTNFRSF21 in EC are still unclear. In this study, we demonstrated that highly expressed circTNFRSF21 in EC are linked to rapid EC cell growth, proliferation and in vivo tumor formation. Mechanically, we showed that circTNFRSF21 could bind to and inhibit miR-1227 activity which could further promote miR-1227 targeted gene MAPK13 expression and MAPK13 downstream gene ATF2 activation. Thus, newly identified circTNFRSF21-miR1227- MAPK13/ATF2 signaling pathway could be potential targets for EC treatment.

## RESULTS

### Characterize of circTNFRSF21 in EC cells

**Figure 1:**
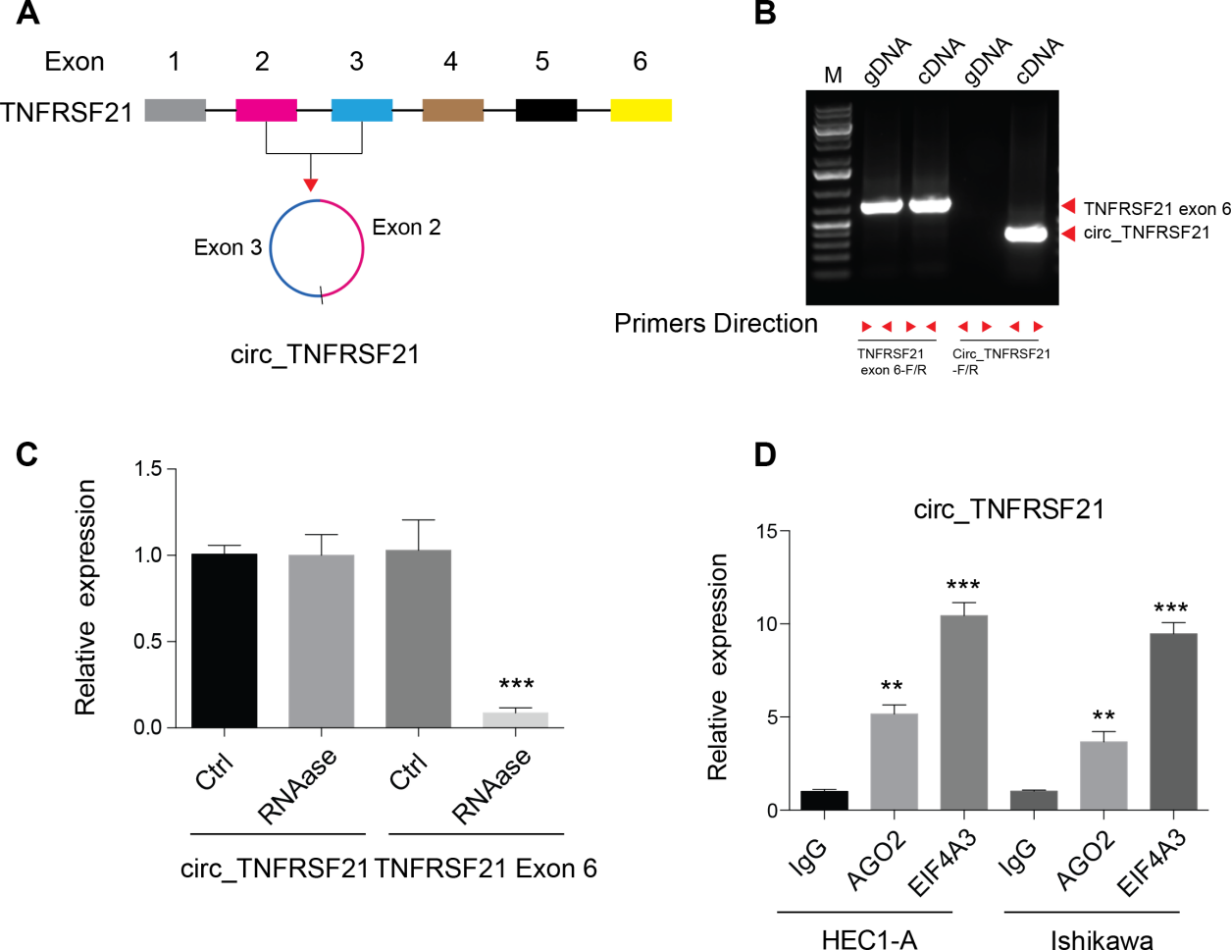
**Detection of circ_TNFRSF21 expression in HEC1-A.** (**A**) Diagram of circ_TNFRSF21. (**B**) PCR results of TNFRSF21 exon 6 or circ_TNFRSF21 from cDNA or genomic DNA (gDNA) (**C**) Detection of circ_TNFRSF21 and TNFRSF21 exon 6 expression before and after RNase A treatment. (**D**) Pull down circ_TNFRSF21 with AGO2 or EIF4A, IgG was used as negative control. *P<0.05, **P<0.01, ***P<0.001, data are represented as mean +/- SEM.

Previous studies showed that circular RNA hsa_circ_0001610 derived from TNFRSF21 was highly expressed in grade 3 EC than their adjacent normal tissues [17]. By studying the circular RNA database, we found that this circular RNA was back spliced by exon 2 and 3 of TNFRSF21 transcripts (Figure 1A). To confirm the existence of circTNFRSF21 in EC cells, Convergent and divergent primers were used to amplify DNA from genomic DNA (gDNA) and cDNA. As shown in Figure 1B, convergent primers can amplify DNA from both gDNA and cDNA, while desired DNA fragment can only be amplified with divergent primers when using cDNA as template. Sanger sequencing confirmed the back spliced junction of TNFRSF21 exon 2 and 3 amplified from EC (Supplementary Figure 1A). Due to its circular structure, circRNA is highly resistance to RNase A degradation. To confirm this, RNA was extracted from EC cells and treated with or without RNase A. qRT-PCR was applied to detect circTNFRSF21 expression. Indeed, RNase A treatment greatly decreased the amount of linear RNA amplified from TNFRSF21 exon 6, with no effect on circTNFRSF21 expression (Figure 1C, ***P<0.001). Ago2 and EIF4A3 are RNA binding proteins. Our bioinformatic analysis showed that circTNFRSF21 has many potential binding sites on Ago2 and EIF4A3. We next asked whether circTNFRSF21 can bind to Ago2 and EIF4A3 in EC. RIP assay with antibodies against Ago2 and EIF4A3 were performed. As indicated, both Ago2 and EIF4A3 greatly precipitated circTNFRSF21 compared with IgG control, and EIF4A3 has a better affinity with circTNFRSF21 than Ago2 (Figure 1D, **P<0.01, ***P<0.001). These results strongly confirmed the existence of circTNFRSF21 in EC cells.

### circTNFRSF21 inhibits TNFRSF21 mRNA and protein expression

**Figure 2:**
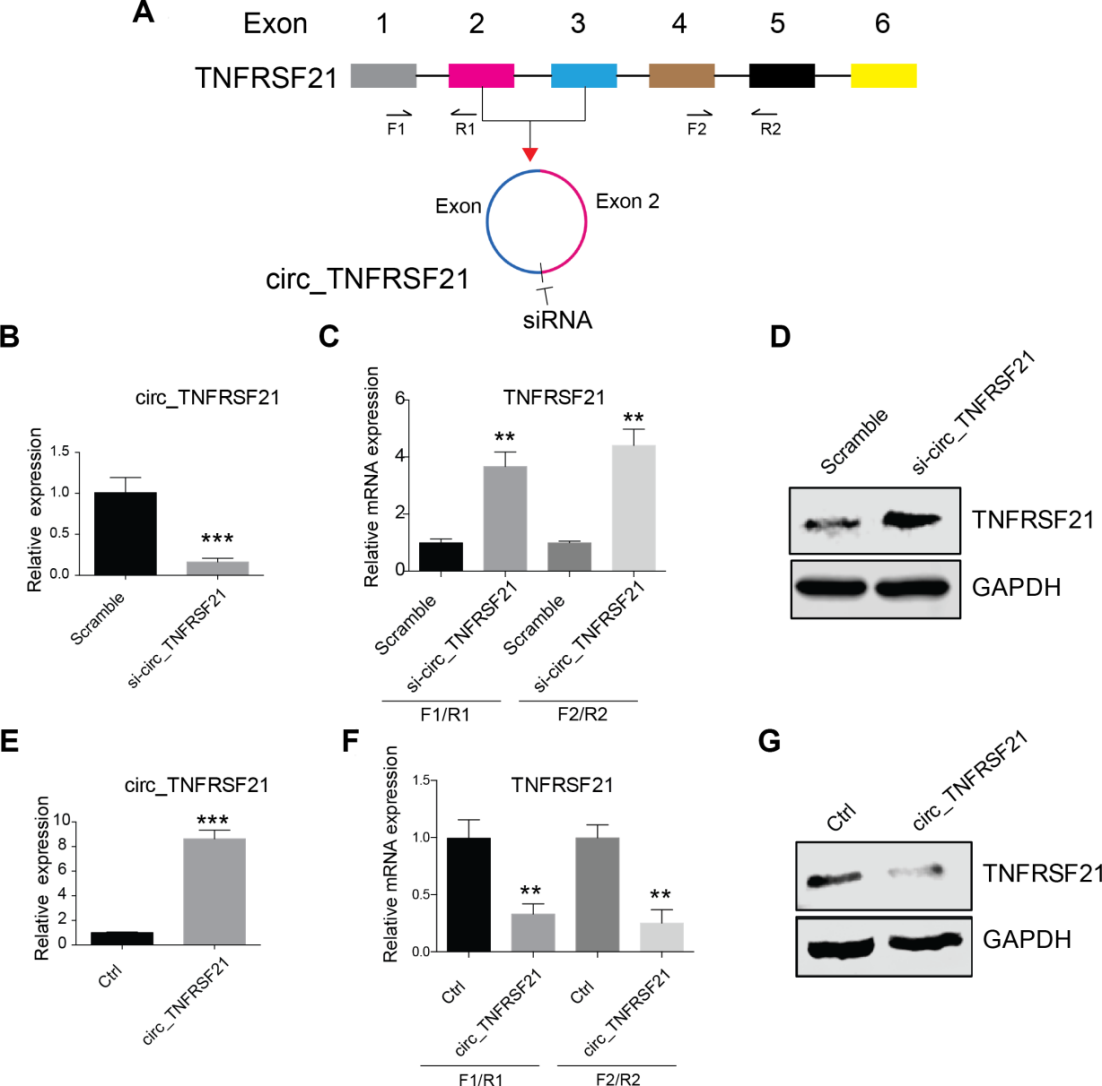
**circTNFRSF21 inhibits TNFRSF21 expression.** (**A**) siRNA targeting the junction of the covalently joined 3' and 5' ends of circ_TNFRSF21 was shown. F1/R1 and F2/R2 were used to amplify TNFRSF21 mRNA. (**B**) Detection of circ_TNFRSF21 expression in HEC1-A after silencing circ_TNFRSF21. TNFRSF21 mRNA (**C**) and protein level (**D**) after silencing circ_TNFRSF21 in HEC1-A. Overexpression of circ_TNFRSF21 (**E**) significantly decreased TNFRSF21 transcription (**F**) and protein synthesis (**G**). *P<0.05, **P<0.01, ***P<0.001. Data are represented as mean +/- SEM.

CircRNA biogenesis may compete with pre-mRNA splicing, which in turn affect mRNA maturation and protein expression (16). We next asked if circTNFRSF21 expressed in EC cells could affect TNFRSF21 mRNA transcription. siRNA targeting the junction of the covalently joined 3' and 5' ends (Figure 2A) was used to knock down circTNFRSF21 in EC cells. As indicated, siRNAs efficiently knocked down circTNFRSF21(Figure 2B and Supplementary Figure 1, ***P<0.001), however, TNFRSF21 mRNA and protein level were greatly upregulated. (Figure 2C–2D, **P<0.01, ***P<0.001). To confirm these results, circTNFRSF21 exon 2, 3 and flanking introns were PCR amplified and inserted into circR vector. CircR vector was constructed by cloning the upstream and downstream intron fragments of MLLT3/AF9 into the EcoRI/EcoRV and EcoRV/XhoI (reverse complement of above) sites of pcDNA3.1. circTNFRSF21 overexpressing plasmid was then introduced into EC cells, the expression of circTNFRSF21, TNFRSF21 mRNA and TNFRSF21 protein were tested. In contrast to circTNFRSF21 knockdown, circTNFRSF21 overexpression (Figure 2E, ***P<0.001) strongly downregulated TNFRSF21 mRNA and TNFRSF21 protein level (Figure 2F, 2G, **P<0.01).

### circTNFRSF21 promotes EC cell growth, proliferation and in vivo tumor formation

**Figure 3:**
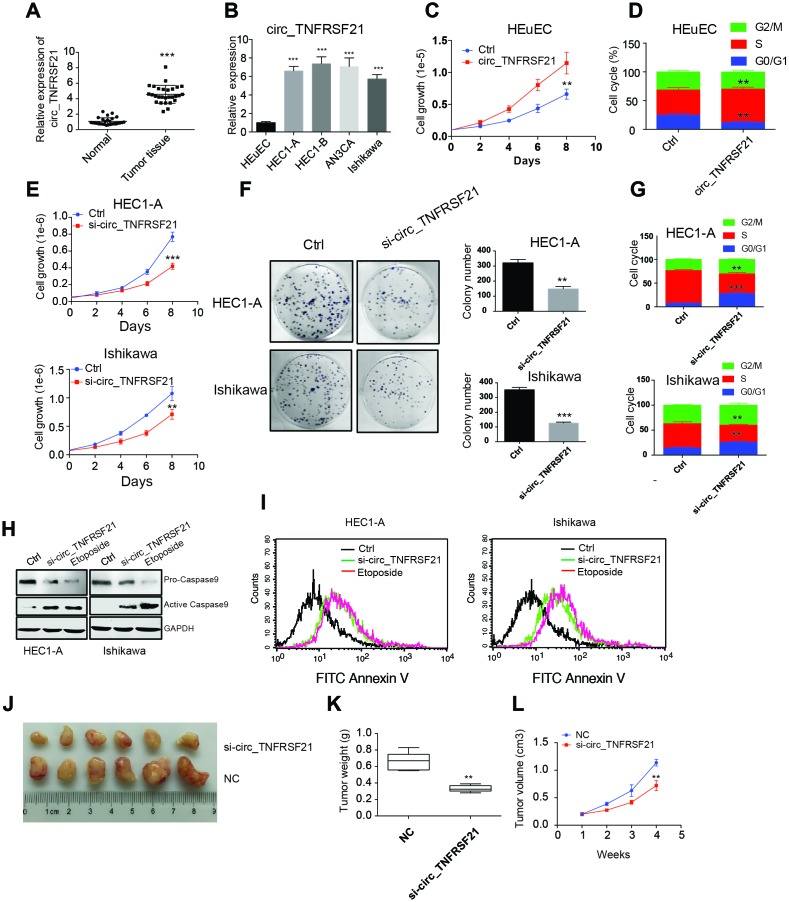
**circTNFRSF21 promotes EC cell growth, proliferation and in vivo tumor formation.** (**A**) circTNFRSF21 expression in EC patient tumor tissues and adjacent normal tissues. (**B**) circTNFRSF21 expression in EC cell lines and HEuEC cells. Cell growth (**C**) and cell cycle (**D**) detection after overexpressing circTNFRSF21 in HEuEC cells. (**E**) Cell growth after silencing circTNFRSF21 in HEC1-A and Ishikawa cells. (**F**) Cell colony formation after silencing circTNFRSF21 in HEC1-A and Ishikawa cells. Statistical results of colony number were shown on the right. (**G**) Cell cycle and (**H**, **I**) Cell apoptosis after silencing circTNFRSF21 in HEC1-A and Ishikawa cells. Etoposide induced cell apoptosis was used as positive control. (**J**) Image of tumors derived from si-circTNFRSF21 and control Ishikawa cells in nude mice (n=6). (**K**) Tumor weights and (**L**) growth curve of tumors from the 2 groups. *P<0.05, **P<0.01, ***P<0.001. Data are represented as mean +/- SEM.

To further understand the roles of circTNFRSF21 in EC. circTNFRSF21 expression in EC patients, EC cell lines HEC1-A, HEC1-B, AN3CA, Ishikawa and normal human endometrial epithelial cells HEuEC were examined. As expected, circTNFRSF21 was greatly upregulated in both EC tumor tissues and EC cell lines compared with their adjacent normal tissues or normal HEuEC cells (Figure 3A, 3B **P<0.01, ***P<0.001). Due to the slightly different expression level of circTNFRSF21among all the four EC cell lines, we randomly selected HEC1-A and Ishikawa for further study. We first tested if highly expressed circTNFRSF21 in EC cells were linked to rapid cell growth. circTNFRSF21 overexpressing plasmid was transfected into HEuEC cells, cell growth and cell cycle were evaluated. Interestingly, overexpressing circTNFRSF21 greatly increased HEuEC cell growth (Figure 3C, **P<0.01), and promoted HEuEC cells transition from G0/G1 to S phage (Figure 3D, **P<0.01). However, knocking down circTNFRSF21 significantly decreased EC cell growth, colony formation, cell cycle progression and increased EC cell apoptosis (Figure 3E–3I, **P<0.01, ***P<0.001).

The role of circTNFRSF21 in tumorigenicity was also examined. Ishikawa cells were subcutaneously injected into the flanks of nude mice in order to yield tumors. After tumors became clearly obvious, siRNA targeting circTNFRSF21 or control siRNA were injected into tumors. Tumor growth was monitored over a 28-day course. As indicated, circTNFRSF21 knockdown greatly impaired tumor growth compared with control group. (Figure 3J–3L, **P<0.01, ***P<0.001). Thus, these results strongly suggested that circTNFRSF21 plays a critical role in promoting EC cell growth and tumor formation.

### TNFRSF21 does not play a role in EC cell growth

**Figure 4:**
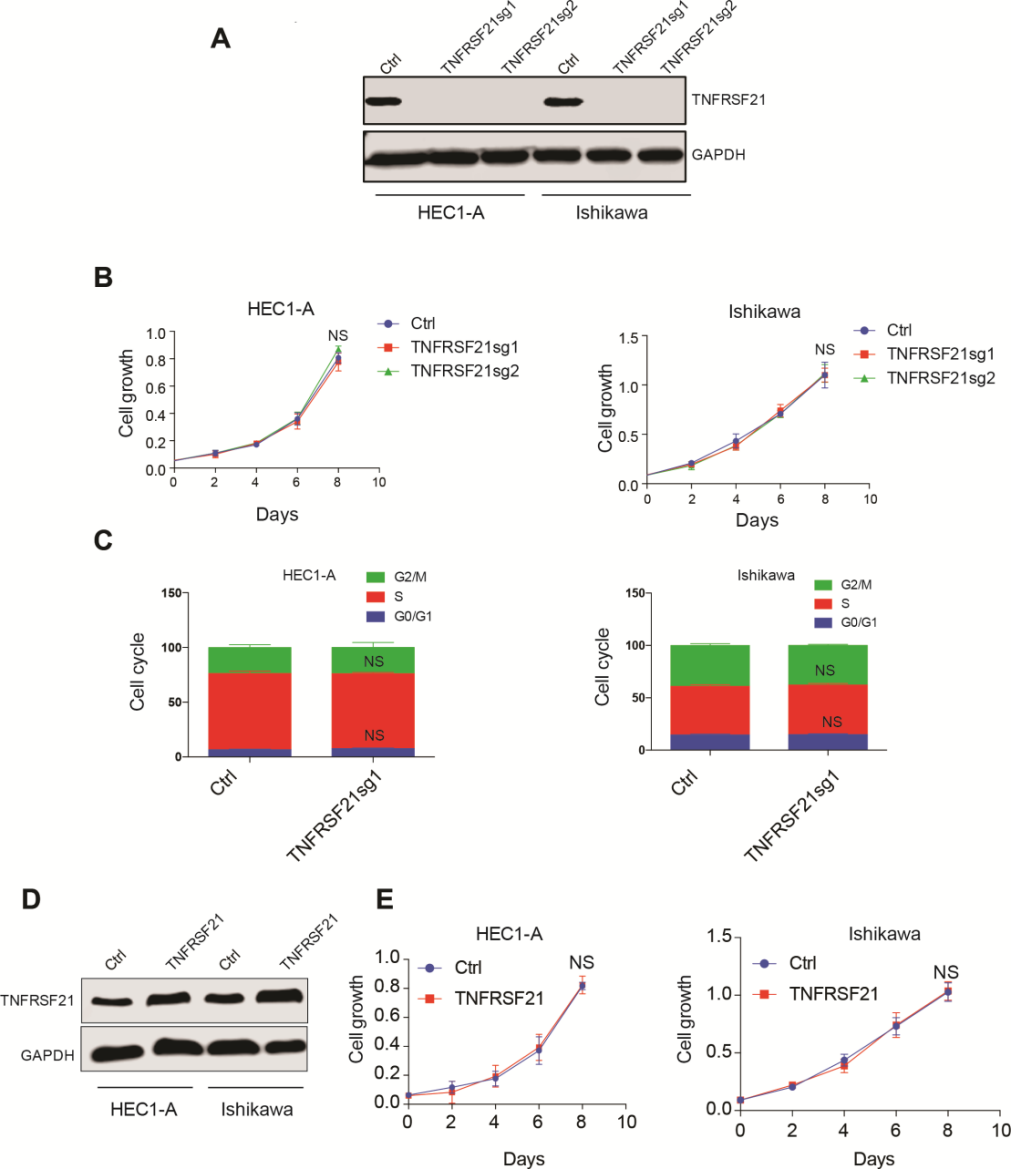
**Knock down or overexpress TNFRSF21 does not affect EC cell growth.** (**A**) Western blot results of TNFRSF21 after knocking down TNFRSF21 in HEC1-A and Ishikawa cells. GAPDH was used as an internal control. Cell growth (**B**) and cell cycle (**C**) detection after knocking down TNFRSF21 in HEC1-A and Ishikawa cells. (**D**) Western blot results of TNFRSF21 after overexpressing TNFRSF21 in HEC1-A and Ishikawa cells. (**E**) Cell growth after overexpressing TNFRSF21 in HEC1-A and Ishikawa cells. Data are represented as mean +/- SEM.

circTNFRSF21 downregulates TNFRSF21 mRNA transcription and protein expression. We next asked if circTNFRSF21 promotes EC cell growth through downregulating TNFRSF21. CRISPR was used to knock out TNFRSF21 in HEC1-A and Ishikawa cells in which Cas9 were stably expressed. TNFRSF21 was efficiently knocked out by Cas9 (Figure 4A). However, cell growth and cell cycle were unaffected even though TNFRSF21 protein level was heavily impaired (Figure 4B, 4C). In line with this, TNFRSF21 overexpression does not affect EC cell growth (Figure 4D, 4E). These results indicated that circTNFRSF21 promotes EC cell growth is not mediated through downregulating TNFRSF21.

### circTNFRSF21 acts as a sponge of miR-1227 in vivo

**Figure 5:**
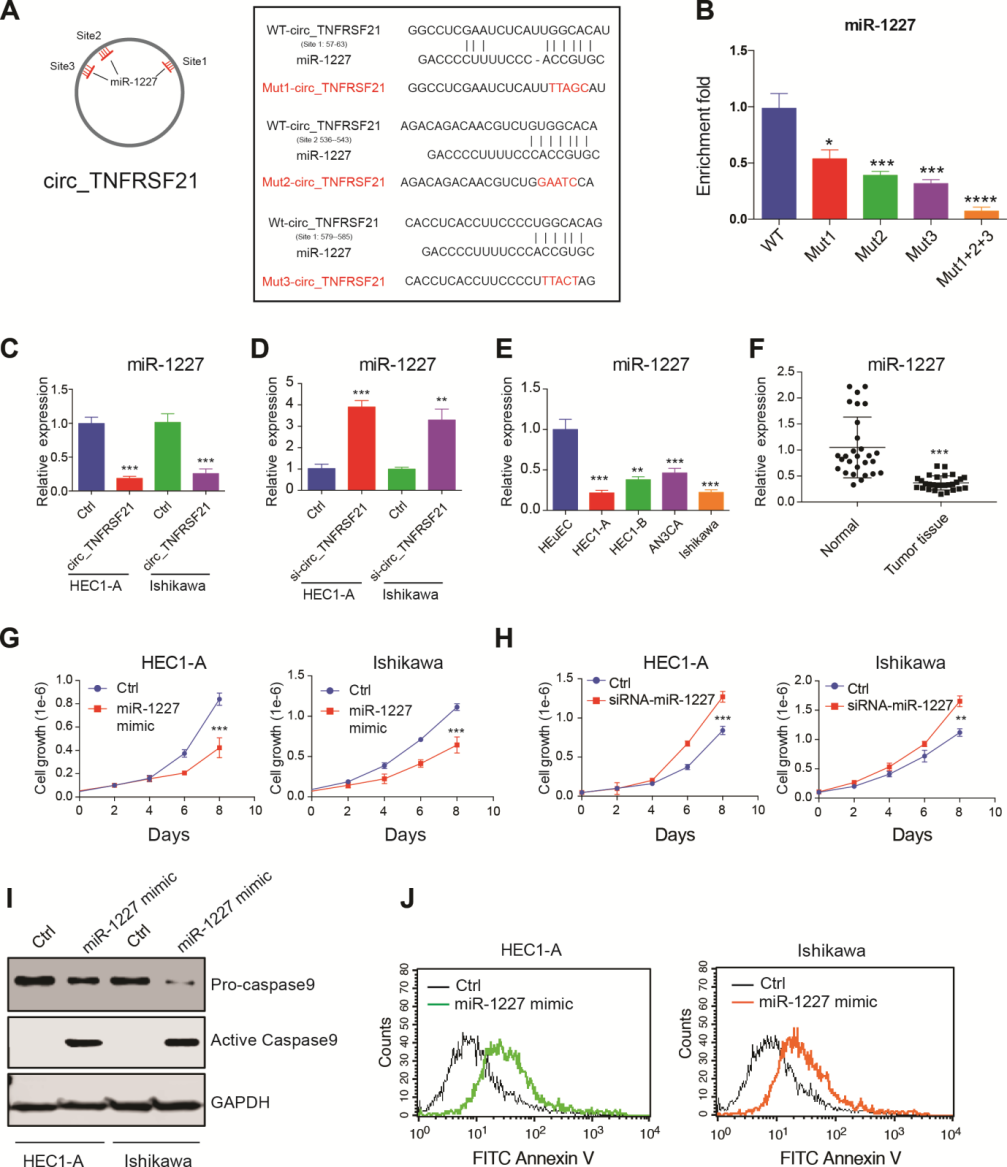
**circTNFRSF21 acts as a sponge of miR-1227 in vivo.** (**A**) Diagram of circ_TNFRSF21 and miR-1227 interactions (**B**) Pull down miR-1227 with biotin labeled WT circTNFRSF21 or miR-1227 binding sites mutated circ_TNFRSF21 from HEC1-A cell lysis. Detection of miR-1227 expression after overexpressing (**C**) or knocking down (**D**) circTNFRSF21 in HEC1-A and Ishikawa cells. (**E**) qRT-PCR detect miR-1227 expression in EC cell lines and HEuEC. (**F**) miR-1227 expression in in EC patient tumor tissues and adjacent normal tissues. (**G**) Cell growth after overexpressing miR-1227 in HEC1-A and Ishikawa cells. (**H**) Cell growth after silencing miR-1227 in HEC1-A and Ishikawa cells. (**I**, **J**) Cell apoptosis detection after silencing miR-1227 in HEC1-A and Ishikawa cells. *P<0.05, **P<0.01, ***P<0.001. Data are represented as mean +/- SEM.

Many studies suggested that circRNA could function as miRNA sponge and regulate miRNA expression [18]. Therefore, we hypothesis that circTNFRSF21 promotes EC cell growth might through working as miRNA sponge. Circular RNA database circinteractome was used to find potential circTNFRSF21-miRNA interactions. Results showed that miR-1227 which has 3 binding sites on circTNFRSF21 is a potential target of circTNFRSF21. The potential miR-1227 binding sites and sequences on circTNFRSF21 were shown in Figure 5A. To confirm miR-1227- circTNFRSF21 interaction is indeed exist within cells, WT circTNFRSF21 or miR-1227 binding sites mutated circTNFRSF21 were labeled with biotin and used for RNA pull down. As indicated, mutating either of these three miR-1227 binding sites significantly decreased circTNFRSF21-miR-1227 interaction. Moreover, the circTNFRSF21-miR-1227 interaction was almost completely abolished when all the three miR-1227 binding sites were mutated. (Figure 5B, *P<0.05, ***P<0.001, ****P<0.0001). We next asked if circTNFRSF21 could affect miR-1227 expression. circTNFRSF21 expressing vector or siRNA vector targeting circTNFRSF21 were exogenously introduced into EC cell lines HEC1-A and Ishikawa, qRT-PCR was then used to detect miR-1227 expression. Results showed that overexpression of circTNFRSF21 significantly decreased miR-1227 expression (Figure 5C, ***P<0.001), while knock down of circTNFRSF21 recovered miR-1227 level in both HEC1-A and Ishikawa cells (Figure 5D, **P<0.01, ***P<0.001).

### miR-1227 affects EC cell growth and apoptosis

As a target of circTNFRSF21, we next asked if miR-1227 plays a role in EC. The expression of miR-1227 in EC cell lines and tumor tissues were first interrogated. Interestingly, miR-1227 expression was significantly lower in EC cell lines and tumor tissues compared with HEuEC and normal tissues respectively (Figure 5E, 5F **P<0.01,***P<0.001). Correlation analysis showed that circTNFRSF21 expression was negatively correlated with miR-1227 expression, confirming circTNFRSF21downregulates miR-1227 in EC (Supplementary Figure 1). The roles of miR-1227 in EC were also examined. As shown in Figure 5G, overexpression of miR-1227 greatly decreased EC cell growth. However, miR-1227 knockdown significantly promoted EC cell growth (Figure 5H, ***P<0.001). The effect of miR-1227 on inducing EC cell apoptosis was also tested. In line with decreased cell growth, miR-1227 mimic also promoted EC cell apoptosis (Figure 5I, 5J). Thus, these results suggested that suppressed miR-1227 expression in EC cells are beneficial for EC cell growth.

### miR-1227 targets MAPK13 and inhibits EC cell proliferation

**Figure 6:**
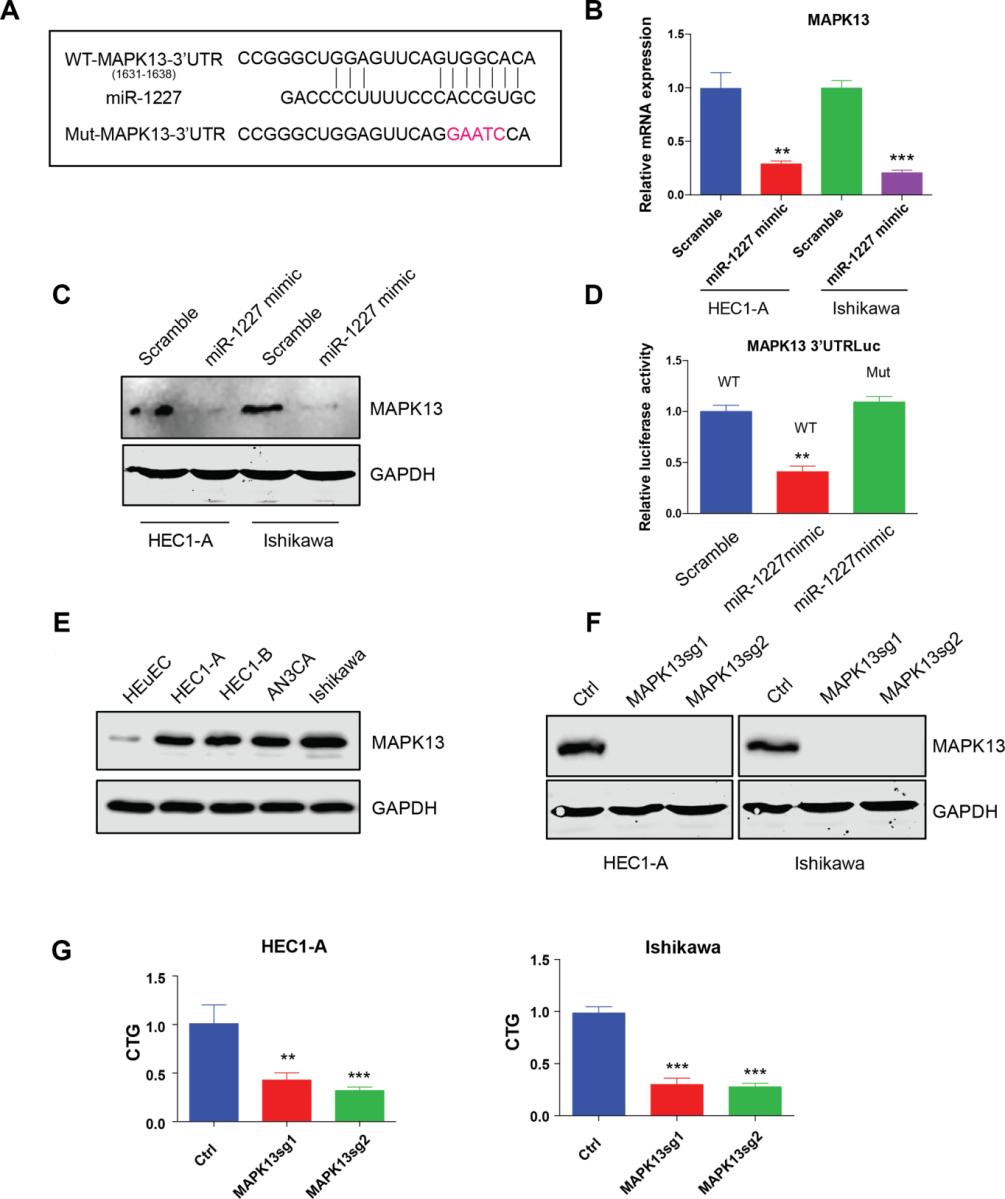
**miR-1227 targets MAPK13 and inhibits EC cell proliferation.** (**A**) Predicted MAPK13 3’UTR-miR-1227 interaction. Detection of MAPK13 mRNA transcription (**B**) and protein level (**C**) after overexpressing miR-1227 mimic in HEC1-A and Ishikawa cells. (**D**) Luciferase assay detects WT or miR-1227 binding sites mutated MAPK13 3’UTR activity in the presence or absence of miR-1227. (**E**) Western blot detects MAPK13 expression in EC cell lines and HEuEC. (**F**) Western blot results of MAPK13 after knocking down MAPK13 in HEC1-A and Ishikawa cells. GAPDH was used as internal control. (**G**) CellTiter-Glo detect cell viability after knocking down MAPK13 in HEC1-A and Ishikawa cells, cell viability was tested 5 days after transduction. *P<0.05, **P<0.01, ***P<0.001. Data are represented as mean +/- SEM.

The most studied roles of miRNA were to impair mRNA stability and further affect protein synthesis. Bioinformatic analysis was then applied to predict miR-1227 targets. Interestingly, MAPK13, a member of the mitogen-activated protein (MAP) kinase family was indicated a potential target of miR-1227. The binding sites of miR-1227 on MAPK13 3’UTR was shown (Figure 6A). To verify MAPK13 is the target of miR-1227 in EC cells, miR-1227 mimic or scramble control was introduced into HEC1-A and Ishikawa cells, the expression of MAPK13 was then tested. Results showed that miR-1227 mimic heavily impaired MAPK13 transcription and protein synthesis, as shown by decreased mRNA and protein level in the presence of miR-1227 mimic (Figure 6B, 6C, **P<0.01, ***P<0.001). In addition, when transfecting miR-1227 mimic and luciferase vector containing wild type or miR-1227 binding sites mutated MAPK13 3’UTR into cells, miR-1227 significantly suppressed WT MAPK13 3’UTR activity with no effect on mutated MAPK13 3’UTR activity (Figure 6D, **P<0.01). These results strongly suggested that MAPK13 is the target of miR-1227 in EC cells. Previous studies on cancer stem-like cells (CSCs) discovered that knock down of MAPK13 greatly abrogated CSCs tumor-initiating ability [19], indicating that MAPK13 might play a role in tumor progression. The roles of MAPK13 in EC cell growth was then investigated. Results suggested that MAPK13 expression was at a higher level in EC cell lines than HEuEC (Figure 6E). CRISPR-Cas9 was used to knock down MAPK13 in HEC1-A and Ishikawa cells in which Cas9 were stably expressed, cell growth was then tested. As indicated, sgRNAs targeting MAPK13 efficiently knocked down MAPK13 (Figure 6F), cell growth was also significantly decreased in the absence of MAPK13 in both HEC1-A and Ishikawa cells (Figure 6G, **P<0.01, ***P<0.001).

### circTNFRSF21 rescues MAPK13-ATF2 signaling pathway activity by acting as miR-1227 sponge in EC cells

**Figure 7:**
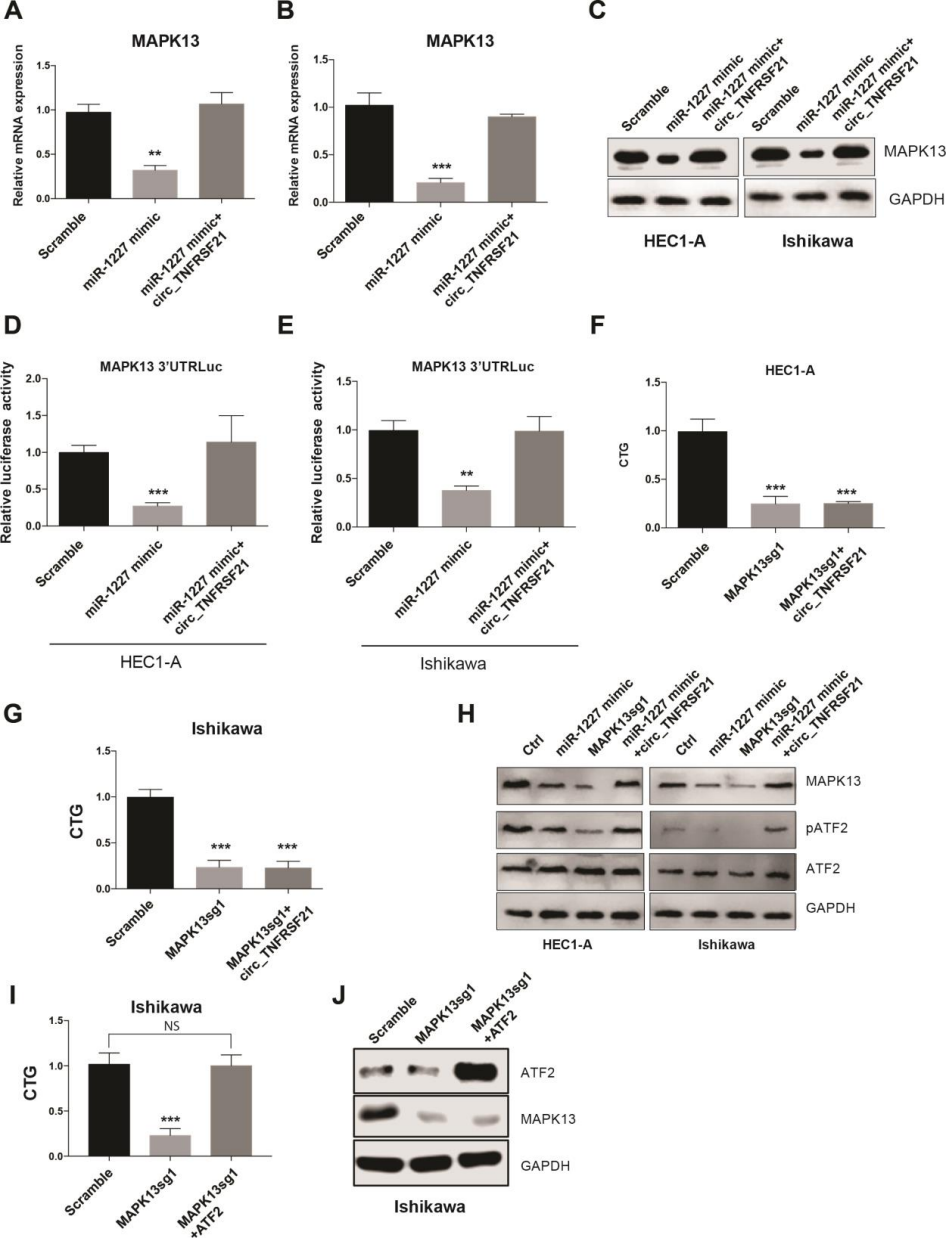
**circTNFRSF21 rescues MAPK13-ATF2 signaling pathway by acting as miR-1227 sponge in EC cells.** Detection of MAPK13 transcription (**A**, **B**) and protein level (**C**). (**D**, **E**) Luciferase assay detect MAPK13 3’UTR activity. (**F**, **G**) Cell growth detection after knocking down MAPK13 in the presence or absence or circTNFRSF21. (**H**) Western blot detects MAPK13, ATF2, phosphorylated ATF2 expression. GAPDH was used as internal control. (**I**) Cell growth detection after knocking down MAPK13 in the presence or absence or ATF2. (**J**) Western blot detects MAPK13, ATF2 expression, GAPDH was used as internal control. *P<0.05, **P<0.01, ***P<0.001. Data are represented as mean +/- SEM.

As miR-1227 sponge, we next asked if circTNFRSF21 could rescue MAPK13 expression impaired by miR-1227. HEC1-A and Ishikawa cells were transfected with miR-1227 in the presence or absence of circTNFRSF21. As expected, miR-1227 greatly downregulated MAPK13 transcription and protein level in both HEC1-A and Ishikawa cells, while presence of circTNFRSF21 almost completely rescued MAPK13 expression (Figure 7A–7C, **P<0.01, ***P<0.001). Luciferase assay with MAPK13 3’UTR showed that circTNFRSF21 restored MAPK13 3’UTR activity suppressed by miR-1227 (Figure 7D, 7E, **P<0.01, ***P<0.001). To find out if MAPK13 is the downstream driver gene for EC cell growth, CRISPR-Cas9 was used to delete MAPK13 in the presence or absence of circTNFRSF21, cell growth was measured 5 days after transfection. Results showed that circTNFRSF21 could not rescue HEC1-A and Ishikawa cell growth after deleting MAPK13 (Figure 7F, 7G, ***P<0.001), indicating MAPK13 is the downstream gene regulated by circTNFRSF21. As a protein kinase, MAPK13 phosphorylate ATF2 is important for cell growth and proliferation. The roles of circTNFRSF21 in MAPK13-ATF2 signaling pathway was also investigated. Indeed, knock down of MAPK13 or over-expressing miR-1227 greatly downregulated MAPK13 expression and phosphorylated form of ATF2 (pATF2), while, circTNFRSF21 rescued MAPK13 and pATF2 expression impaired by miR-1227 (Figure 7H). To further confirm ATF2 is the downstream gene regulated by MAPK13. ATF2 was introduced into EC cells followed by MAPK13 knockout. As expected, MAPK13 knockout greatly decreased EC cells growth. However, ATF2 restored EC cell growth impaired by MAPK13 knockout (Figure 7I–7J), These results further confirmed that ATF2 is the downstream gene regulated by MAPK13 in EC cells.

## DISCUSSION

Despite great advances in cancer treatment, surgery remains a mainstay of treatment for most women with endometrial cancer [20]. Although molecular targeted therapies including Anti-angiogenic agents, epidermal growth factor receptor (EGFR) inhibitors, HER2/neu antibodies, and phosphoinositide 3-kinase (PI3K)-PTEN-AKT-mammalian target of rapamycin (mTOR) pathway inhibitors showed promising efficiency in EC treatment, the underlying molecular mechanism of EC development remain largely unknown [18, 21, 22]. In this study, for the first time, we showed that circTNFRSF21, a newly identified circular RNA can promote EC cell growth through competitively interact with miR-1227 to modulate MAPK13 expression, these results provided new insights into the molecular basis of EC.

Rapid development of next generation sequencing facilitated the discovery of novel circular RNA [23–25]. CircRNA as a newly identified non-coding RNA has been reported to play critical roles in cancer development. For example, circNHSL1 was reported to promote gastric cancer progression through acting as a miR-1306-3p sponge to relieve the repressive effect of miR-1306-3p on its target SIX1 [26]. N6-methyladenosine (m6A) modified circNSUN2 was frequently upregulated in tumor tissues and serum samples from colorectal carcinoma patients with liver metastasis and predicts poorer patient survival [27]. Previous genome-wide circular RNA sequencing with grade 3 endometrial cancer tissues and adjacent tissues found that hsa_circ_0001610 was upregulated over 18 folds in cancer tissues compared with normal tissues [17]. However, the biological property and function of hsa_circ_0001610 in EC is not studied. In this study, we first showed that hsa_circ_0001610 was derived from TNFRSF21 by back splicing on TNFRSF21 exon 2 and 3. Overexpression of circTNFRSF21 was also confirmed in EC tumor tissues and EC cells compared with normal adjacent tissues and normal human endometrial epithelial cells. Functional studies showed that knock down of circTNFRSF21 greatly suppressed EC cell growth, cell colony formation and cell cycle progression, indicating upregulated circTNFRSF21 played an important role in promoting EC cell survival and proliferation.

Previous studies suggested that many circular RNAs can compete with its own pre-mRNA splicing and affect mRNA production. In this studied we showed that silencing circTNFRSF21 greatly upregulated TNFRSF21 mRNA transcription and protein expression. In addition, exogenous overexpress circTNFRSF21 suppressed TNFRSF21 mRNA and protein level. Thus, circTNFRSF21 belongs to a type of circular RNA whose expression could compete with its own mRNA synthesis. However, whether circTNFRSF21 could affect other gene transcription needs more investigation.

An increasing number of studies showed that circRNA may function as a sponge of miRNA and affect miRNA targeted gene expression. For example, circTRIM33-12 was shown to suppress hepatocellular carcinoma progression by acting as the sponge of MicroRNA-191 [28]. circLARP4 acts as a sponge of miR-761 and promotes RUNX3/p53/p21 expression, which further induces cellular senescence in hepatocellular carcinoma [19]. In this study, by using bioinformatics analysis we found that miR1227 which have 3 predicated binding sites on circTNFRSF21 is a potential circTNFRSF21 target. RNA immunoprecipitation confirmed their directly interaction in vivo. Either knock down or overexpress circTNFRSF21 significantly affected miR-1227 expression, indicating that miR-1227 is the target of circTNFRSF21 in EC. Previous studies on characterizing miRNA expression profiles in normal and osteoarthritic human chondrocytes showed that miR-1227 was upregulated in normal chondrocytes. Consistent with this, in this study we found that miR-1227 expression level was much higher in normal human endometrial epithelial cells than in EC cells. Functional studies showed that overexpression of miR-1227 greatly suppressed EC cell growth. Thus, targeting miR-1227 may provide advantages for EC treatment.

MAPK13 belongs to a member of the mitogen-activated protein (MAP) kinase family [29]. Like other MAPKs, MAPK13 plays an important role in cellular responses evoked by extracellular stimuli leading to direct activation of transcription factors such as ATF2 [30]. MAPK13 activation is involved in a wide variety of cellular processes such as proliferation, differentiation, transcription regulation and cancer development [19, 31–33]. In this study we found that miR-1227 targets MAPK13 in EC cells, overexpressing miR-1227 significantly downregulated MAPK13 expression and MAPK13 3’UTR activity. Knock down MAPK13 greatly impaired EC cell growth and cell cycle progression, indicating MAPK13 is essential for EC cell survival. ATF2 also known as cyclic AMP response element binding protein 2 and CRE-BP1 is a member of the activating protein-1 (AP1) transcription factor family. Dysregulation of ATF2 has been reported in cancer development including synovial sarcomas, as well as in prostate and head and neck squamous cancers [34–38]. In this study we found that miR-1227 mediated MAPK13 downregulation also impaired ATF2 activation. However, the presence of circTNFRSF21 almost completely restored both MAPK13 expression and ATF2 activity impaired by miR-1227. Thus, these results strongly support the hypothesis that circTNFRSF21 facilitates MAPK13/ATF2 signaling pathway activation by acting as a competing endogenous RNA (ceRNA) of miR-1227. circTNFRSF21-miR-1227-MAPK13/ATF2 axis are potential targets for EC treatment in the future.

## CONCLUSIONS

In this study we demonstrated that circTNFRSF21 was highly expressed in EC patients and EC cells, and highly expressed circTNFRSF21 promoted EC cell growth, cell cycle progression and in vivo tumor formation. Mechanically, we found that circTNFRSF21 could function as a sponge of miR-1227 and inhibit miR-1227 mediated MAPK13/ATF2 signaling pathway suppression. Thus, this study suggested that circTNFRSF21-miR-1227-MAPK13/ATF2 axis is a promising target in EC treatment in the future.

## MATERIALS AND METHODS

### Cell lines

Endometrial cancer cell lines HEC1-A, HEC1-B, AN3CA, Ishikawa and normal human endometrial epithelial cells HEuEC were used for this study. HEC1-A, HEC1-B, AN3CA were purchased from ATCC, Ishikawa was obtained from Shanghai Institute of Biosciences Cell Resource Center, Chinese Academy of Sciences. HEuEC was purchased from Lifeline Cell Technology, 293T cells were purchased from ATCC, Fetal bovine serum (FBS) was purchased from Gibco. HEC1-A was maintained in McCoy's 5a Medium added with 10% FBS, HEC1-B and AN3CA were cultured in Eagle's Minimum Essential Medium supplemented with 10% FBS, Ishikawa was cultured in MEM + 2mM Glutamine + 1% Non Essential Amino Acids + 5% Fetal Bovine Serum. HEuEC was maintained in Lifeline’s ReproLife™ Medium. 293T cells were cultured in DMEM medium supplemented with 10% FBS. All cell lines were maintained at 37°C with 5% CO_2_.

### Patient samples

28 EC patients from Beijing Friendship Hospital were enrolled in this study. All the EC and adjacent normal endometrial tissues were collected during surgery and rapidly frozen down in liquid nitrogen and stored at -80°C for future use. The characteristics of the EC patients were listed in Table 1. This study was approved by the Ethics Committee of the Beijing Friendship Hospital Affiliated to Capital Medical University. All patients signed consent forms.

**Table 1:** The characteristics of the EC patients.

**circTNFRSF21**			**miR-1227**		
	**High (n=16)**	**Low (n=12)**	**P value**	**High (n=9)**	**Low (n=19)**
Characteristics				
Age					
>= 50	11(68.75%)	8 (66.6)	0.907	5 (55.55%)	12(63.15%)
<50	5 (31.25%)	4 (33.3)		4(44.45%)	7(36.85%)
Lymphatic metastasis					
Yes	10(62.5%)	3(25%)	0.049*	3 (33.33%)	14 (73.68)
NO	6(37.5)	9(75%)		6(66.66%)	5 (26.31)
FIGO stage					
I-II	9(56.25%)	2(16.6%)	0.033*	6 (66.66%)	7 (36.84%)
III-IV	7(43.75%)	10(83.4%)		3 (33.33%)	12 (63.16%)
Histological grade					
G1	5(31.25%)	9 (75%)	0.022*	7 (77.77%)	6(31.58%)
G2+G3	11 (68.75)	3(25%)		2 (22.23)	13(68.42%)
Menstruation					
Non-menopause	2(12.5%)	7(58.33)	0.012*	7 (77.77%)	5 (26.31%)
Menopause	14 (87.5%)	5 (41.66)		2 (22.23%)	14 (73.68)

### RNA extraction and qRT-PCR

RNA was extracted by using TRIzol. Extracted RNA was reverse transcript into cDNA by using iScript™ Reverse Transcription Reagents (Bio-rad). SYBR master mix was used to determine RNA expression by real-time PCR analyses. U6 was used to normalize miR-1227 expression. GAPDH (Glyceraldehyde 3-phosphate dehydrogenase) was used as an internal control for circular RNA and mRNA expression. RNA expression level was calculated with 2-ΔΔct. Primers used were listed in Supplementary Table 1.

### RNase a treatment

RNA was extracted with TRIzol and dissolved in 20 μl TE buffer. RNA was then treated with 2 μl RNase A (10 mg/ml, Fermentas) and incubate for 1h at 37°C before reverse transcription.

### RNA immunoprecipitation (RIP)

Magna RIP RNA-Binding Protein Immunoprecipitation Kit (Millipore, Bedford, MA) was used to pull down RNA-protein complexes. Briefly, cells were trypsin digested, washed with PBS and lysed with lysis buffer. After lysis RNA-protein complexes were pulled down by using antibodies of interest parallel with control IgG. Proteins were then degraded with proteinase K and RNA was then extracted. Targeted RNA was detected by qRT-PCR.

### Oligonucleotide transfection

siRNA targeting circTNFRSF21, miR-1227 mimic and scramble control were synthesized in Genewiz (Shanghai, China). Lipofectamine RNAiMax (Life Technologies) was used to transfect cells according to manufacturer’s instructions.

### Reporter assay

Q5 Site-Directed Mutagenesis kit (NEB, USA) was used to generate mutant MAPK13 3'-untranslated region (3'-UTR) in which miR-1227 binding sites were mutated. Wild-type and mutant MAPK13 3’UTR were cloned into the pGL3-control vector. Lipofectamine 2000 (Invitrogen; Thermo Fisher Scientific, Inc.) was used to transfect plasmids into cells according to manufactures instructions. Dual-Luciferase reporter assay kit (Promega Corporation, USA) was used to determine luciferase activity.

### Western blot

Western blot was used to determine protein level. In brief, cells were harvested and lysed with cell lysis buffer (10 mM Tris, pH 7.4. 100 mM NaCl. 1 mM EDTA. 1 mM EGTA. 1% Triton X-100. 10% glycerol. 0.1% SDS. 0.5% deoxycholate.) containing protein inhibitors. Protein was extracted and quantitated by BSA assay. Same amount of protein was loaded into SDS-PAGE (sodium dodecyl sulphate-polyacrylamide gel electrophoresis) gel and separated by electrophoresis. Separated protein was transferred to nitrocellulose membrane at 100V for 1h. Membrane was then blocked with 5% non-fat milk. Primary antibody targeting protein of interest was incubated with membrane at 4°C with shaking overnight. HRP tagged secondary antibodies were used to probe protein of interest. Odyssey CLx was used to capture images.

### Cell growth assay

Cell growth was measured by cell counting or by CellTiter-Glo (CTG) luminescent assay. In brief, 24h after transfection, cells were trypsin digested and seeded into 6 well plates at a concentration of approximately 0.1million cells per ml. Cell numbers were recorded at days 2,4,6 and 8 after cell outgrowth. For cell growth tested with CellTiter-Glo (CTG) luminescent, cells were harvested at day 5 post cell growth, relative cell number was detected with CTG reagent according to manufacturer’s instructions. cell viability was recorded with Luminometer.

### Propidium iodide (PI) staining

Propidium iodide (PI) stain was used to detect cell cycle. Cells were harvested, washed with ice cold PBS and re-suspended in 100μl ice cold PBS. Cell fixation was performed by adding 900μl cold 100% ethanol into cell suspension and incubate overnight at 4°C. The next day, cells were washed with PBS twice and 1ml PI stain solution (50 μg/ml; 1 mg/ml of RNase A, 0.1% Triton X-100 in PBS) was used to stain cells at room temperature for 30 to 60 mins before applied to FACS sorting detection.

### Cell apoptosis detection

Cell Apoptosis was detected with FITC Annexin V Apoptosis Detection kit II purchased from BD Pharmingen™. Briefly, cells were washed with ice cold PBS twice and then resuspended in 1X Binding Buffer at a concentration of 1 x 10^6^ cells/ml. 100 μl of cell suspension was transferred into a 5 ml culture tube and cells were stained with 5 μl of FITC Annexin V by incubating in the dark at RT for 15 min. After staining, 400 μl of 1X Binding Buffer was added into cell solution, cell apoptosis was detected by FACS.

### Cell colony formation

After transfecting cells for 24h cells were harvested and counted. 300 cells were re-seed into 6-well plates. Cells were allowed to outgrowth for another 2-3 weeks. Once cell colonies are clearly observed, medium were removed, and 500ul methanol was used to fix cells. Colony was stained with 0.1% crystal violet at room temperature for another 5 minutes. Colony numbers were recorded, representative images were captured by scanner.

### Biotin pull down assay

Biotin was used to label fragment containing either wild type circTNFRSF21 or miR-1227 binding sites mutated circTNFRSF21 and conjugated to streptavidin magnetic beads. Cell lysis was then incubated with probe coated beads with shaking at 4°C overnight. The next day, beads were washed, and RNA was eluted with elution buffer. miR-1227 was quantified by qRT-PCR.

### CRISPR-Cas9 mediated gene knock out

SgRNAs targeting TNFRSF21 and MAPK13 were designed with online tools (https://www.benchling.com/), and oligos were synthesized in Sangon, shanghai. Paired sgRNAs were annealed and inserted into pLentiGuide-puro vector and packaged into 293T cell with PsPAX2 and VSVG. Lentiviruses were harvested 48h after transduction and were used to infect Cas9 stable cell line HEC1-A-cas9 and Ishikawa-cas9. Uninfected cells were eliminated by selecting with puromycin for 3 days. Western blot was used to determine the efficiency of gene knockout.

### In vivo tumor formation assays

Animal research was conducted in accordance with the recommendations in the Guidelines for the Care and Use of Laboratory Animals of China strictly. All efforts were made to minimize suffering. Twelve female BALB/c nude mice (5 weeks of age, ~20 g weight) were purchased from Hua Dong normal university animal center (Shanghai, China). After one-week adaption in our animal center, six mice were subcutaneously injected with Ishikawa cells at a concentration of 5×10^6^ cells/200 μl in sterile saline. When tumor volume reaches approximately 30 mm^3^ at 8 days later, 1μg control or siRNA targeting circ_TNFRSF21 were injected into tumor at days 0, 3 and 7. The volume of tumor was assessed every week until the end of the experiment. The tumor weight was determined after the animals were sacrificed. This study was approved by the Animal Ethics Committee of the Beijing Friendship Hospital Affiliated to Capital Medical University

### Bioinformatics

TargetScan was applied to search genes targeted by miR-1227. Circinteractome was used to predict potential circTNFRSF21-miRNA interactions.

### Statistical analysis

Statistical analysis was performed using Prism 6. The differences between experimental group and control group was tested by Student's t test. All data were shown as mean ± SEM. *P < 0.05, **P<0.01, ***P<0.001. P < 0.05 was considered statistically significant.

## Supplementary Material

Supplementary Figure 1

Supplementary Table 1
