# Supplementary material for: circTNFRSF21, a newly identified circular RNA promotes endometrial carcinoma pathogenesis through regulating miR-1227-MAPK13/ATF2 axis
Source: Aging (Albany NY). 2020 Apr 16;12(8):6774–92. doi: 10.18632/aging.103037 (PMC7202486; doi:10.18632/aging.103037)
Supplement: Supplementary Figure 1 [file aging-12-103037-s002..pdf]

SUPPLEMENTARY FIGURE

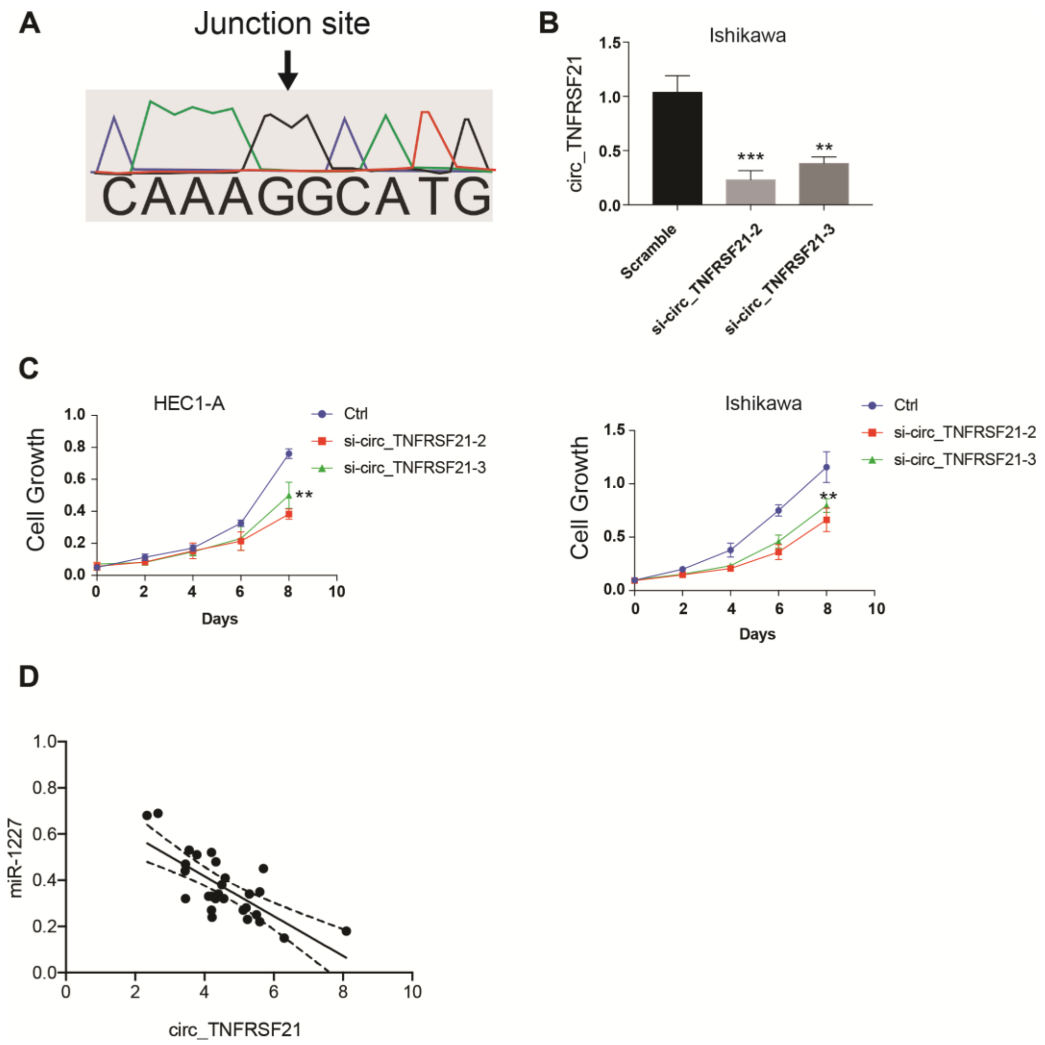

Supplementary Figure 1. (**A**) Sanger sequencing results of circ_TNFRSF21. The junction site was indicated with black arrow. (**B**) Two siRNAs targeting circ_TNFRSF21 greatly decreased circ_TNFRSF21 expression. (**C**) EC cell growth after silencing circ_TNFRSF21 by using siRNAs. (**D**) The correlation analysis of circ_TNFRSF21 and miR-1227 expression in EC patients.
