# Supplementary material for: circTNFRSF21, a newly identified circular RNA promotes endometrial carcinoma pathogenesis through regulating miR-1227-MAPK13/ATF2 axis
Source: Aging (Albany NY). 2020 Apr 16;12(8):6774–92. doi: 10.18632/aging.103037 (PMC7202486; doi:10.18632/aging.103037)
Supplement: Supplementary Table 1 [file aging-12-103037-s001..pdf]

SUPPLEMENTARY TABLE

Supplementary Table 1. Primers used in this study.

MAPK13sg1-F	caccgTCCAGGAGCCCAATGACCTG
MAPK13sg2-F	caccgACAGCTCGGCCATCGACAAG
MAPK13sg1-R	aaacCAGGTCATTGGGCTCCTGGAc
MAPK13sg2-R	aaacCTTGTCGATGGCCGAGCTGTc
TNFRSF21sg1-F	caccgACCTTTGGGAACATAAGTGG
TNFRSF21sg2-F	caccgACTTGTCACAGGTTAGCACC
TNFRSF21sg1-R	aaacCCACTTATGTTCCCAAAGGTc
TNFRSF21sg2-R	aaacGGTGCTAACCTGTGACAAGTc
TNFRSF21 exon6-F	CCTGACCTGCTGTAGAACATAG
TNFRSF21 exon6-R	CTCCAGCCCAGAGCATATTAG
Circ_TNFRSF21-F	GACATAGGTTCCTGCTGGACAC
Circ_TNFRSF21-R	CGCCCATCAAGGGCCCCAAG
qRT-MAPK13-F	TGAGCCGACCCTTTCAGTC
qRT-MAPK13-R	AGCCCAATGACGTTCTCATGC
Mut-Circ_TNFRSF21-F1	GAATCTCATTttagcATACCGCCATGTTGACC
Mut-Circ_TNFRSF21-R1	GAGGCCTTCTGTTCTGGC
Mut-Circ_TNFRSF21-F2	ACAACGTCTGgaatcACTCCCGTCCTTCTCCAG
Mut-Circ_TNFRSF21-R2	CTGTCTCCTTGGTCCCCG
Mut-Circ_TNFRSF21-F3	ACCTTCCCCTttactAGCCATCTTTCCACG
Mut-Circ_TNFRSF21-R3	GAGGTGGAGCTGGAGAAG
Mut-MAPK13-3'UTR-F	TGGAGTTCAGgaatcCAATTACATCTTACTGCAACCTCAGCCTCC
Mut-MAPK13-3'UTR-R	GCCCGGGTGACAGAGCCA
MAPK13 3'UTR-F	gggcacagtgggtctcaaatt
MAPK13 3'UTR-R	gcctataatcccagcactttgg
qRT-Circ_TNFRSF21-F	TGCCAATGAGATTCGAGGC
qRT-Circ_TNFRSF21-R	TTGTGGAAAAGGCAGGGC
GAPDH-F	GTAGAGCGGCCGCCATG
GAPDH-R	GATTTCCATTGATGACAAGC
qRT-U6-F	TTGGTCTGATCTGGCACATATAC
qRT-U6-R	AAAAATATGGAGCGCTTCACG
qRT-TNFRSF21-F1	tccccggttcagccatgggg
qRT-TNFRSF21-R1	tctgttctggctgagctgtg
qRT-TNFRSF21-F2	tagcgccctg cgccagcacc
qRT-TNFRSF21-R2	ggctaagcgggctggggctca
miR-1227 mimic	GACCCCUUUUCCCACCGUGC
miR-1227 Scramble	CTGACACUTCAGACCCCCCTA
